# Under Conditions of Amyloid Formation Bovine Carbonic Anhydrase B Undergoes Fragmentation by Acid Hydrolysis

**DOI:** 10.3390/biom11111608

**Published:** 2021-10-30

**Authors:** Victor Marchenkov, Natalya Ryabova, Vitaly Balobanov, Anatoly Glukhov, Nelly Ilyina, Natalya Katina

**Affiliations:** Institute of Protein Research RAS, 142290 Pushchino, Russia; march@phys.protres.ru (V.M.); ryabova@phys.protres.ru (N.R.); balobanov@phys.protres.ru (V.B.); gluktol@gmail.com (A.G.); nelly.ilyina@mail.ru (N.I.)

**Keywords:** amyloid, carbonic anhydrase, acid hydrolysis, size-exclusion chromatography, peptides

## Abstract

The development of many severe human diseases is associated with the formation of amyloid fibrils. Most of the available information on the process of amyloid formation has been obtained from studies of small proteins and peptides, wherein the features of complex proteins’ aggregation remain insufficiently investigated. Our work aimed to research the amyloid aggregation of a large model protein, bovine carbonic anhydrase B (BCAB). It has previously been demonstrated that, when exposed to an acidic pH and elevated temperature, this protein forms amyloid fibrils. Here, we show that, under these conditions and before amyloid formation, BCAB undergoes fragmentation by acid hydrolysis to give free individual peptides and associated peptides. Fragments in associates contain a pronounced secondary structure and act as the main precursor of amyloid fibrils, wherein free peptides adopt mostly unstructured conformation and form predominantly irregular globular aggregates. Reduced acidity decreases the extent of acid hydrolysis, causing BCAB to form amorphous aggregates lacking the thioflavin T binding β-structure. The presented results provide new information on BCAB amyloid formation and show the importance of protein integrity control when working even in mildly acidic conditions.

## 1. Introduction

Protein misfolding and the formation of amyloid aggregates are causes of severe diseases which are currently considered incurable. More than fifty diseases are attributed to amyloidosis, including Alzheimer’s disease, Parkinson’s disease, diabetes type II, and other disorders [1,2]. Some of them are associated with the aggregation of peptides that result from the proteolysis of larger precursor proteins [3,4]. However, the formation of amyloids is not always a pathological process. In some organisms, amyloid fibrils are of functional importance [5,6]. In recent years, amyloids have been studied as potential biomaterials due to their high stability and mechanical strength [7].

The intensive development of solid-state NMR methods, X-ray diffraction, and cryo-electron microscopy has made it possible to obtain information on the molecular structure of amyloid fibrils formed by certain proteins [8,9,10,11,12]. A specific feature of amyloid fibrils is the cross-β-structure with β-strands aligned perpendicular to the aggregate axis, and hydrogen bonds between strands are parallel to the axis [8,9]. Compared to the β-structure of globular proteins, the amyloid cross-β-structure shows a higher regularity and slighter twist [13,14].

Dobson’s group made a significant contribution to the understanding of the fundamental laws of amyloid aggregation. They showed that, apart from proteins forming amyloids in vivo, there are polypeptide chains which can form fibrils under appropriate solvent conditions [15,16]. Based on the data obtained, it was concluded that forming amyloid fibrils is specific, not only for disease-associated proteins. Furthermore, it is instead a common property of all polypeptide chains [15]. These results led to an intensive study of amyloid aggregation using model proteins as examples [17,18,19].

It has been shown that the destabilization of protein structure and the presence of non-native conformations in solution are the conditions required for aggregation [20,21,22,23]. Therefore, amyloid aggregation is usually studied under denaturing conditions, such as high temperatures and low pH [16,24]. Investigations of the amyloid formation showed that the aggregation rate depends on the physicochemical properties of amino acid residues, such as hydrophobicity, charge, and β-propensity [25,26]. Then, these parameters formed a basis for theoretical predictions of the effect of amino acid substitutions on the rate of amyloid aggregation [27,28].

However, the majority of information on amyloid formation comes from studies involving small proteins or peptides [1,25]. Therefore, one of the further research areas of amyloid aggregation is revealing the specific features of amyloidogenesis by complex proteins. Our work aimed to investigate the aggregation of a large model protein, bovine carbonic anhydrase B (BCAB), which serves as a convenient model with a well-described folding [29,30,31]. Because BCAB is one of a few proteins forming cytotoxic fibrils, identifying the specificities of this protein aggregation could help us to understand the mechanism of cell damage in amyloidosis [32].

BCAB is a single-domain protein consisting of 260 amino acid residues. It is a metalloenzyme that catalyzes the hydration of carbon dioxide (CO_2_) to the bicarbonate ion (HCO_3_^−^) [33]. The secondary structure of the native state of BCAB is composed of a twisted β-sheet, which is antiparallel mainly, two β-hairpins, and short α-helices [34]. The folding of this protein proceeds through two intermediate states: the pre-molten globule and the molten globule [29].

BCAB has been shown to form amyloid fibrils under acidic conditions, that is, at pH = 2.4 and *t* = 57 °C [35,36]. As the pH rises to 3.6, the formation of amorphous aggregates is observed [35]. Based on the experimental data on the aggregation of BCAB and the peptide corresponding to amino acid residues 201–227, a conclusion on differences in amyloid formation by the full-length protein and its fragment has been drawn [36].

In this work, we show for the first time that, under amyloid formation conditions, BCAB is susceptible to acid hydrolysis. The hydrolysis products are both unfolded free peptides and peptides as associates containing pronounced secondary structures. The data obtained concluded that the precursors of BCAB amyloids are the associated peptides. At the same time, free peptides form predominantly globular aggregates that do not contain the thioflavin T binding β-structure. Our results on the amyloid formation accompanied by the fragmentation of the precursor protein can help us to understand the mechanism of in vivo aggregation of proteolysis-yielded peptides.

## 2. Materials and Methods

### 2.1. BCAB Expression and Purification

The plasmid pET-11c_joe encoding BCAB was expressed in *E. coli* BL21DE3 cells at 37 °C. According to the early method, the protein was purified by anion exchange chromatography and gel-filtration [32]. In this work, the apo-form of BCAB was used. Bound zinc ions were removed by dialysis in 25 mM MOPS, pH 7.0, containing 100 mM dipicolinic acid.

### 2.2. BCAB Aggregation Conditions

BCAB aggregation experiments used 50 mM glycine-HCl buffer (pH 2.7) and 50 mM Na phosphate-citrate buffer (pH 2.7 and 4). After the dissolution of lyophilized BCAB, the solutions were centrifuged at 120,000× *g* for 30 min to remove the formed protein associates. Protein concentration was determined by UV absorption at 280 nm wavelength on a Cary 100 spectrophotometer (Agilent Technologies, Palo Alto, CA, USA). The extinction coefficient ε = 1.72 was calculated theoretically [37]. The concentration of peptides yielded by acid hydrolysis was estimated by the absorption at 245 nm.

For the aggregation study, 4 mg/mL protein solutions were incubated at 40 °C or 57 °C with stirring at 300 rpm in a Biosan TS-100 thermal shaker (Biosan, Riga, Latvia). To obtain aggregates formed at pH 4 (40 °C) and at pH 2.7 (57 °C), the protein solution was centrifuged at 45,000× *g* for 30 min. Then the precipitate was resuspended in a buffer solution of the original volume.

### 2.3. Fluorescent Spectroscopy

Thioflavin T fluorescence spectra were measured using a Varian Cary Eclipse spectrofluorimeter (Agilent Technologies, Palo Alto, CA, USA) at 25 °C. Protein solutions with a concentration of 4 μM (0.12 mg/mL) were used; the dye concentration was 25 μM. Measurements were carried out in a 3 × 3 mm cuvette. The excitation wavelength was 450 nm, the emission spectra were recorded in the range 460–600 nm, and kinetic curves were plotted by fluorescence intensity at the 480 nm emission wavelength.

### 2.4. Electron Microscopy

The protein solution (0.1 mg/mL) was applied onto a formvar-coated copper grid and contrasted with 1% uranyl acetate solution. Micrographs were obtained using a transmission electron microscope JEM 1200 EX (Jeol, Tokyo, Japan) at 80 kV accelerating voltage.

### 2.5. SDS-PAGE

SDS-PAGE was performed by the standard method [38]. The acrylamide concentration in the stacking gel was equal to 5%, a PAAG gradient of 5–20% was used in the separating gel. Dual Xtra Prestained Protein Standards 2–250 kDa (Bio-Rad Laboratories, Inc., Hercules, CA, USA) were used as a standard of molecular weights.

### 2.6. Size-Exclusion Chromatography

Size-exclusion chromatography was performed on a Superdex 200 10/300 GL column (GE Healthcare Bio-Sciences AB, Uppsala, Sweden) attached to the ProStar HPLC chromatographic system (Varian, Walnut Creek, CA, USA). The mobile phase was composed of 50 mM sodium phosphate with citric acid added to pH 2.7. The flow rate was 0.4 mL/min. The volume of the injected sample was 0.2 mL. Protein elution was followed by the photodiode array absorbance detector and a fluorescence detector set to excitation wavelength at 280 nm and emission wavelength at 340 nm.

### 2.7. Circular Dichroism (CD)

CD spectra were measured using a Chirascan spectropolarimeter (Applied Photophysics, London, England). For measurements in the far UV range, a cuvette with 0.1 mm optical path length was used. The protein concentration was 1 mg/mL. All the measurements were carried out at 25 °C. Molar ellipticity [Θ] was calculated as $\left[\Theta \right]=\frac{\Theta_{\lambda }\times MRW}{l\times C}$, where $\Theta_{\lambda }$ is the measured ellipticity value at λ (mgrad); *MRW* is the mean molar weight of the residue calculated from the amino acid sequence, *l* is optical path length (mm), *C* is the protein concentration (mg/mL).

## 3. Results

### 3.1. BCAB Undergoes Hydrolysis under Conditions of Amyloid Formation

**Figure 1:**
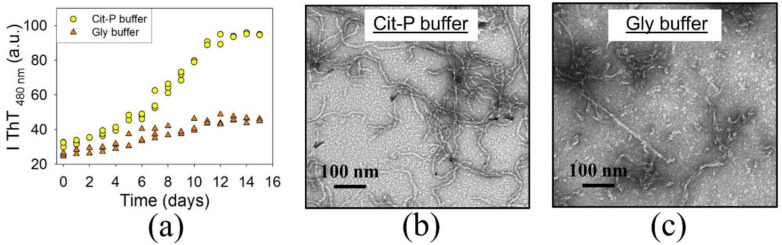
Kinetics of BCAB amyloid formation at pH = 2.7 and 57 °C recorded by thioflavin T fluorescence assay (for each condition three repeated experiments have been carried out) (**a**). Electron micrographs of aggregates formed after 15 days of incubation in citrate-phosphate (Cit-P) (**b**) and glycine (Gly) (**c**) buffer solutions.

It was shown previously that BCAB forms amyloids at acidic pH and an elevated temperature (57 °C, pH = 2.4) in glycine (Gly) buffer [35,36]. Thus, our initial experiments were conducted under similar conditions (57 °C, pH = 2.7). Since protein aggregation may depend on solvent components, we compared amyloid formation by BCAB in two buffer systems keeping pH within the acidic range, in glycine (Gly) and citrate–phosphate (Cit-P) buffer solutions [39]. In both cases, the protein concentration was 4 mg/mL. The aggregation kinetics recorded by thioflavin T fluorescence are presented in Figure 1a. The obtained data show that the fluorescence intensity of aggregates formed in the Cit-P buffer is significantly higher than that of aggregates from the Gly solution.

The morphology of aggregates formed after 15-day incubation of the protein solutions was examined by electron microscopy. According to the micrographs, BCAB forms fibrils in the Cit-P buffer (Figure 1b). On the other hand, in the Gly buffer, the aggregates are predominantly rod-shaped, and only a tiny fraction of them show fibrillar morphology (Figure 1c). Thus, the Cit-P buffer solution appears to be optimal for forming BCAB fibrils, and we used it in our further experiments.

**Figure 2:**
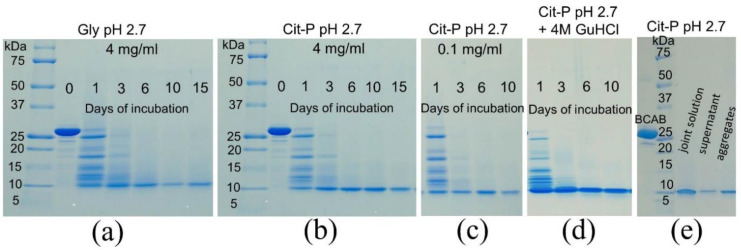
SDS-PAGE electrophoregrams of samples collected during incubation at pH 2.7 and 57 °C. 4 mg/mL BCAB in Gly buffer (**a**); 4 mg/mL BCAB in Cit-P buffer (**b**); 0.1 mg/mL BCAB in Cit-P buffer (**c**); 4 mg/mL BCAB in Cit-P supplemented with 4M GuHCl (**d**). Samples obtained after 15 days of incubation in Cit-P at 4 mg/mL BCAB (**e**). The amount of protein applied was 10 µg per lane.

It is known that incubation of proteins at high temperatures in acidic mixtures can lead to acid hydrolysis of the polypeptide chain [40,41,42,43]. Thus, we analyzed protein integrity by SDS-PAGE during the BCAB aggregation in Gly and Cit-P buffers (Figure 2a,b). Surprisingly, after a one-day incubation of BCAB under acid amyloidogenic conditions, almost the entire protein was cleaved into peptides of 10–25 kDa. During the following five days of incubation, further hydrolysis produced mainly peptides of about 10 kDa. The hydrolysis is buffer-independent since its pattern and kinetics are the same in glycine and citrate-phosphate buffer solutions. Hence, the data allow us to conclude that BCAB undergoes hydrolysis before amyloid formation, and the precursors of amyloid are the peptides that result from cleavage rather than the full-length protein.

According to the literature, there are two major types of acid hydrolysis. In harsh conditions (110 °C, 2–6 M acid), a complete cleavage of the protein chain into amino acids or di-and tri-peptides is observed [40,41]. In milder conditions, at 65–70 °C, limited acid hydrolysis occurs, with the resulting peptides of a certain constant length [42,43]. Our results show that under amyloidogenic conditions, BCAB undergoes limited acid hydrolysis that is almost complete after 6-day incubation (Figure 2b). In this case, the formed amyloid fibrils are composed of ≈10 kDa peptides.

The formation of amyloids occurs at BCAB concentration of 4 mg/mL, while no aggregation is observed when the protein concentration decreases to 0.1 mg/mL. Therefore, we compared hydrolysis at concentrations of 4 mg/mL and 0.1 mg/mL. The cleavage rate and the molecular weight distribution of the resulting peptides were the same (Figure 2b,c). Therefore, the presence of aggregates in the solution does not affect the rate and pattern of BCAB acid hydrolysis.

To test the possibility of protease-induced or structure-dependent autocatalytic fragmentation, a study of protein hydrolysis at pH 2.7 in the presence of 4M guanidine hydrochloride was performed. Under these conditions, almost all polypeptide chains are fully unfolded. The resulting electrophoregram showed rapid cleavage of the protein (Figure 2d). Thus, under these conditions, the protein fragmentation is caused by acid-induced hydrolysis rather than the action of contaminating proteases whose activity would be blocked by the denaturant. The addition of guanidine hydrochloride slightly accelerates the cleavage of BCAB because, after 1-day incubation, the content of peptides of molecular weight above 15 kDa is less than that in the absence of the denaturant (Figure 2b,d). The limited acid hydrolysis is site-specific, and the N-terminal part of serine and the C-terminal side of aspartic acid residues are the most susceptible to cleavage [41,43]. Moreover, it is supposed that basic residues providing local buffering capacity affect certain bond breakdown probabilities [41]. Therefore, a faster degradation of BCAB in the presence of a denaturant may result from the complete unfolding of the protein molecule and reflect the effect of the protein structure on the degradation rate under acid conditions.

To confirm that the amyloids are formed from the hydrolysis products, we centrifuged the solution of the fibrils formed after 15 days of incubation at pH 2.7 and 57 °C. Then the supernatant and the pellet were subjected to SDS-PAGE. The obtained electrophoregram shows that both the supernatant and the large aggregates from the pellet are composed of polypeptide chains with a mass of about 10 kDa each (Figure 2e). Therefore, the BCAB fibrils were dissociated by SDS, and the molecular weight of the polypeptide chains included could be estimated by SDS-PAGE.

### 3.2. The Precursors of BCAB Amyloids Are Associated Peptides

The obtained results put the question as to the precursor of BCAB amyloids. Two possible options can be supposed: (i) the hydrolyzed protein dissociates to free individual peptides that form fibrils, or (ii) after hydrolysis, the polypeptide fragments remain bound in a compact structure, and in this case, the associated peptides are the amyloid precursors. To answer this question, it is necessary to estimate the molecular weights of the species present in the solution after hydrolysis. Because the cleavage of BCAB does not depend on its concentration, the hydrolysis experiments used a concentration of 1 mg/mL, with which no amyloid aggregation occurs.

**Figure 3:**
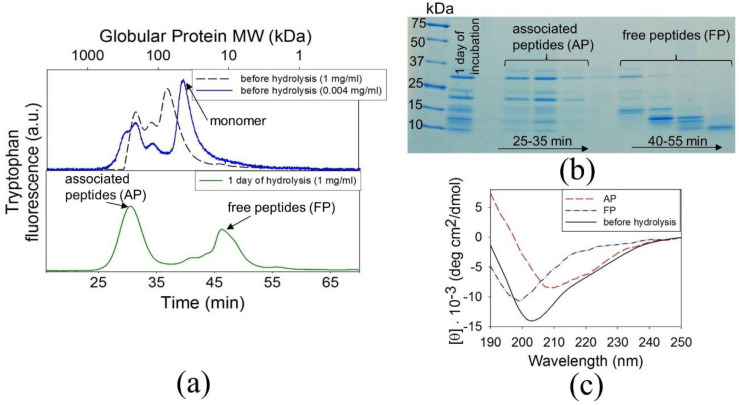
Size-exclusion chromatography of ВСАB at pH 2.7 before (upper panel) and after 1-day hydrolysis (lower panel) (**a**). SDS-PAGE electrophoregram of chromatographic fractions of the sample after 1-day hydrolysis (**b**). Far UV CD spectra of BCAB before hydrolysis at pH 2.7, associated peptides (AP) and free peptides (FP) (**c**).

To determine whether BCAB dissociates into free peptides after hydrolysis, we compared size-exclusion chromatography elution profiles of the protein before and after 1 day of incubation at pH 2.7 and 57 °C (Figure 3a). At pH 2.7, BCAB acquires an intermediate state [44,45]. Therefore, its elution corresponds not to a protein of 29 kDa (BCAB molecular weight) but to a globular protein of a higher molecular weight (≈40 kDa). The elution profile shows the peak of monomeric protein only when the protein is diluted to a concentration of 0.004 mg/mL. In contrast, at a concentration of 1 mg/mL and higher, BCAB is present in the form of associates (Figure 3a). In the elution profile of the sample after 1-day hydrolysis, there are two peaks with the maxima corresponding to 200 kDa and 12 kDa. SDS-PAGE of the obtained fractions indicates that both peaks contain peptides with a wide range of molecular weights (Figure 3b). These findings allow a conclusion that after 1-day hydrolysis, the BCAB solution contains both free isolated peptides (FP) and associated peptides (AP).

The secondary structure of BCAB before hydrolysis, AP, and FP were investigated by the CD in the far UV region (Figure 3c). At pH 2.7 before fragmentation, BCAB has a pronounced secondary structure, as evidenced by molar ellipticity values around 7–8 deg×cm^2^/dmol at 215–222 nm. The CD spectrum of AP shows that after hydrolysis, fragments in associates retain the secondary structure of full-length BCAB. In contrast, the spectrum of FP demonstrates a loss of the secondary structure, as evidenced by a pronounced minimum at 198 nm and the decreased CD bands at 215 and 222 nm.

**Figure 4:**
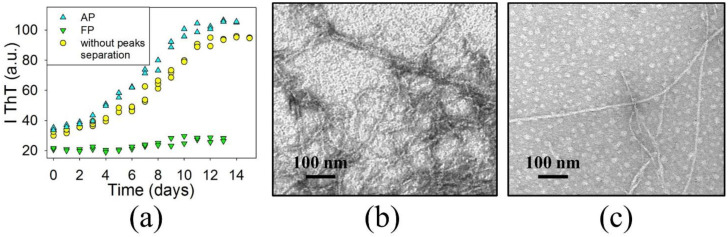
Aggregation kinetics of associated peptides (AP) and free peptides (FP), monitored by thioflavin T fluorescence (two repeated experiments have been carried out) (**a**). Electron micrographs of aggregates formed by AP (**b**) and FP (**c**) after 14 days of incubation.

Thus, the BCAB hydrolysis yielded two fractions: free and associated peptides. To determine which of these the precursors of BCAB amyloids are, we used the following approach. First, AP and FP were separated by gel filtration and concentrated. Then the thioflavin T fluorescence assay was used to test each fraction for its ability to form amyloid aggregates (Figure 4a).

As seen, the kinetic curve of amyloid formation for the BCAB solution without separation into fractions and AP coincide, while FP form aggregates characterized by insignificant dye binding. Furthermore, the electron microscopy results confirm that after 14 days of incubation, AP form fibrillar aggregates (Figure 4b). In contrast, the image of aggregates formed by FP shows predominantly globular aggregates and some fraction of long fibrils (Figure 4c).

Thus, we conclude that, under conditions of amyloid aggregation, BCAB undergoes fragmentation by acid hydrolysis, producing free and associated peptides. The latter retains the secondary structure and acts as the main precursor of amyloid fibrils. At the same time, free peptides adopt mainly an unstructured conformation and form irregular globular aggregates with some fraction of fibrils.

### 3.3. Does Full-Length BCAB Form Aggregates?

Since at pH 2.7 and 57 °C the amyloid formation is preceded by BCAB hydrolysis, one could propose that protein fragmentation is a prerequisite for BCAB amyloidogenesis and that no higher-order aggregates can be formed by full-length protein. To verify this suggestion, we tried to find conditions where BCAB forms amyloids from the full-length chain. Because acid hydrolysis requires both low pH and a high temperature, the protein cleavage can be delayed or completely inhibited in two ways, either pH elevation or/and temperature decrease. Therefore, we studied BCAB aggregation at pH 2.7 and 40 °C, pH 4 and 40 °C, and pH 4 and 57 °C. At pH values above 4.8 BCAB does not dissolve at high concentrations required for amyloid aggregation.

**Figure 5:**
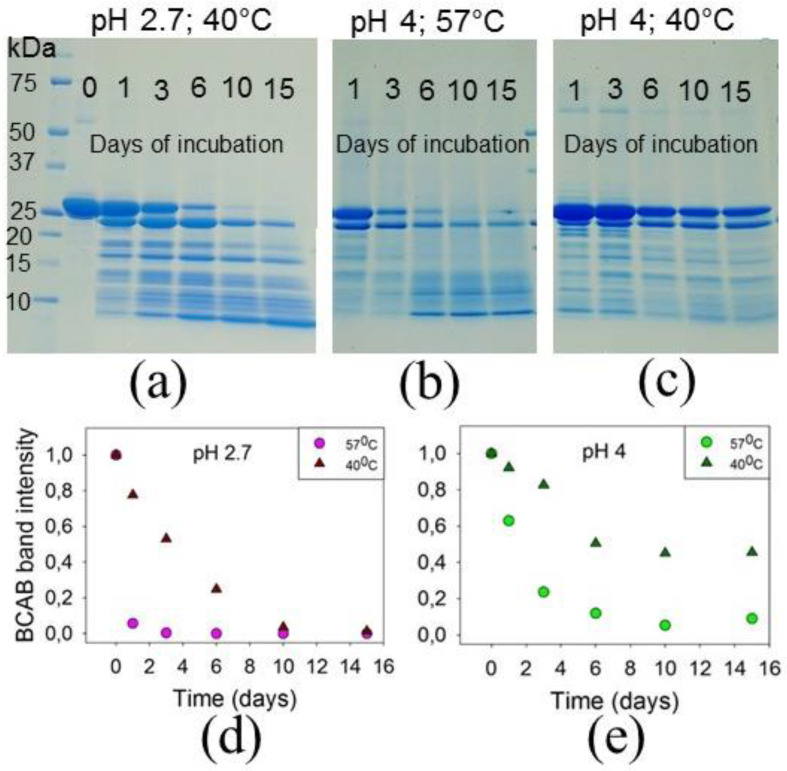
SDS-PAGE electrophoregrams of BCAB aliquots collected during incubation at pH 2.7 and 40 °C (**a**); at pH 4 and 57 °C (**b**); at pH 4 and 40 °C (**c**). Changes in the full-length BCAB band intensity during incubation at pH 2.7 (**d**) and pH 4 (values are normalized relative to the total band intensity of the lane) (**e**).

Figure 5a–c presents SDS-PAGE electrophoregrams of samples collected after different incubation periods under the above conditions. Time dependence graphs of the full-length BCAB band intensity in the gel were plotted (Figure 5d,e). As seen, at pH 2.7 and 40 °C, the protein fragmentation rate was lower than that at pH 2.7 and 57 °C. Surprisingly, at pH 4 at both temperatures studied, a pronounced hydrolysis of the protein was observed, and even at 40 °C, after 6 days of incubation, about 50% of BCAB was cleaved into peptides (Figure 5e).

**Figure 6:**
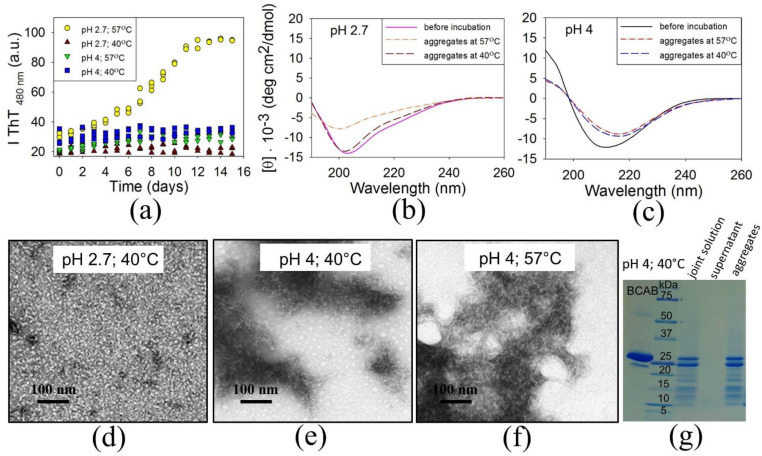
Time dependence of thioflavin T fluorescence after dye addition to BCAB solutions incubated at pH 2.7 and pH 4 at two different temperatures, 57 °C, and 40 °C (for each condition, three repeated experiments have been carried out) (**a**). Far UV CD spectra of BCAB before incubation and after 15 days of incubation at pH 2.7 (**b**) and at pH 4 (**c**). Electron micrographs of aggregates formed by BCAB after incubation during 15 days at pH 2.7 and 40 °C (**d**), at pH 4 and 40 °C (**e**), at pH 4 and 57 °C (**f**). SDS-PAGE electrophoregram of BCAB solution after 15 days of incubation at pH 4 and 40 °C (**g**).

The kinetics of BCAB aggregation under these conditions were investigated using the thioflavin T fluorescence method (Figure 6a). To determine whether changes in the secondary structure occur during the aggregation, the CD spectra of the solutions before and after 15 days of incubation under all studied conditions were measured (Figure 6b,c). Figure 6a shows that dye-binding aggregates are formed only at pH 2.7 and temperature of 57 °C. The electron micrograph reveals fibrils under these conditions (Figure 1b). Despite the decrease in CD signal in the spectrum of aggregates formed at pH 2.7 and 57 °C in comparison with that of the protein before incubation, the shapes of both spectra are similar, thus indicating that no significant changes occur in the secondary structure during amyloid aggregation (Figure 6b). This is confirmed by the calculation of the secondary structure content using the BeStSel service that gives values 26.2% of the β-structure and 25.6% of α-helices in BCAB before incubation versus 30.7% of the β-structure and 17.2% of α-helices in the amyloid aggregates [46]. Thus, the question arises as to what causes the increase in the ThT intensity during amyloid formation. According to published data, the thioflavin T binds to the cross-β-structure with five or more β-strands of a flat β-sheet. This structure is a feature of regular amyloids, while the monomeric proteins contain a predominantly twisted β-structure that does not bind the dye [14]. This suggests that a significant increase in the ThT fluorescence intensity during BCAB amyloid aggregation results not from the formation of an additional β-structure but rather from repacking the present β-structure, making it a more regular one.

In other conditions studied, no significant increase in the thioflavin T fluorescence intensity was observed (Figure 6a). But the electron microscopy images evidence that the types of aggregates formed differ significantly (Figure 6d–f). At pH 2.7 and 40 °C, BCAB forms small globular oligomers with a diameter of about 5 nm and with the secondary structure similar to that of a monomeric protein (Figure 6b,d). Large aggregates do not emerge, possibly because hydrophobic interactions are not stable enough under these conditions. At pH 4 before and after incubation at both temperatures studied, BCAB has a pronounced β-structure, as evidenced by the presence of a minimum at 210–215 nm at the CD spectra (Figure 6c). However, the protein forms amorphous and globular aggregates under these conditions without a periodic β-structure binding thioflavin T (Figure 6a,e,f).

Only pH 4 and 40 °C are the conditions that preserve the full-length BCAB after hydrolysis (Figure 5c). To determine whether the aggregates were formed by full-length protein, we had the solution centrifuged after 15 days of incubation. Then the pellet was resuspended in the buffer, and both the pellet and supernatant were subjected to SDS-PAGE (Figure 6g). The electrophoregram clearly shows that the full-length protein and peptides form aggregates, while the supernatant does not contain polypeptide chains. Thus, we can conclude that with an increased pH, full-length BCAB is incorporated into massive amorphous aggregates that do not bind thioflavin T.

The fibrils formation requires denaturing conditions for proteins that acquire non-native conformations with hydrophobic groups exposed to a solvent. However, amyloidogenesis demands not only intermolecular hydrophobic interactions but also the formation of a regular cross-β-structure by the protein molecules. For BCAB, it is shown that pH 4, where the protein is not entirely cleaved, provokes the formation of amorphous aggregates that do not bind thioflavin T. Both the full-length protein and peptides are involved in their formation. This suggests that BCAB easily forms intermolecular interactions at higher pH values, but conformational rearrangements with the formation of higher-order amyloid β-structures are impossible or energetically unfavorable.

## 4. Discussion

### 4.1. Precursor of BCAB Amyloids Is Protein Fragments

Since the formation of amyloid fibrils leads to severe human diseases, protein aggregation is intensively studied. However, most of these studies deal with small proteins. The next important step in this direction is studying the mechanism of aggregation of large complex proteins. Our work was aimed at investigating the amyloid formation of BCAB which comprises 260 amino acid residues. It has been shown previously that this protein forms amyloids at acidic pH and an elevated temperature [35,36]. In this work, we demonstrate for the first time that, under these conditions, BCAB undergoes acid hydrolysis with its fragments, rather than the whole protein, being precursors of amyloids.

The formation of amyloids by hydrolysis products is observed not only for BCAB and other proteins under acidic conditions. Fragments resulting from enzymatic proteolysis in vivo are precursors of amyloids associated with human diseases. Alzheimer’s disease is a striking example where amyloids are formed by the Aβ-peptide, a fragment of a large transmembrane protein APP (amyloid precursor protein) [3,47]. Usually, studies of this type of aggregation focus on an individual peptide, a constituent of fibrils. However, the process of amyloid formation by peptides resulting from precursor protein hydrolysis is more complex. Therefore, investigation of the mechanism of aggregation under acidic conditions could be helpful for better understanding the role of protein fragmentation in amyloidosis.

In this work, we have shown that, after BCAB hydrolysis, the solution contains free peptides and peptides retained in associates. The associated peptides are homogeneous in molecular weight, preserve a pronounced secondary structure of the full-length protein, and act as precursors of amyloid fibrils. In addition, free peptides adopt a less structured conformation and form predominantly irregular globular aggregates with a small fraction of fibrils. These new data on amyloid formation by BCAB contribute to our knowledge of the aggregation mechanism of peptides that are proteolysis products.

### 4.2. Acid Hydrolysis of Proteins: Features and the Need to Test

Acid hydrolysis of a polypeptide chain is observed under acidic conditions at high temperatures. Classical acid hydrolysis studies were conducted under harsh solvent conditions (110 °C and 2–6 M acids), resulting in complete protein cleavage into amino acids [40,41]. Limited hydrolysis can yield peptides in milder conditions, at 65 °C and pH below 2 [42,43]. However, because the previous hydrolysis studies were aimed at the cleavage of complete proteins, the chosen experimental conditions provided a relatively fast fragmentation. A further temperature decrease (below 65 °C) and an increase in pH (above 2) decelerate hydrolysis with the possibility of protein degradation still preserved. Amyloid aggregation studies require long-term protein incubation in denaturing conditions because the fibrils formation takes several days or weeks. As a result, the question of the conditions under which the slow hydrolysis of protein chains is possible has become actual, and protein fragmentation by acid hydrolysis has been observed for some proteins during amyloid formation [48,49].

The results of the current work show that, despite the lower rate and degree of acid hydrolysis, the protein undergoes cleavage over a wide pH and temperature range, including pH 4 and 40 °C. A pronounced degradation of the proteins under these mild conditions seems unexpected, since this temperature is within the physiological range. Therefore, our results indicate the importance of the protein chain integrity control during incubation under acidic conditions, even slightly increasing temperature. We believe that the presented results will be helpful for experiments requiring the long-term incubation of proteins under acidic conditions, including the study of amyloid aggregation.
